# The Effect of Pellicle on Biofilm Formation in a Supragingival Biofilm Model

**DOI:** 10.1002/cre2.70276

**Published:** 2025-12-29

**Authors:** Shengjile Deari, Monika Gothwal, Kay Gränicher, Thomas Thurnheer, Thomas Attin, Lamprini Karygianni

**Affiliations:** ^1^ Clinic of Conservative and Preventive Dentistry, Center of Dental Medicine University of Zurich Zurich Switzerland

**Keywords:** biofilm model, colony‐forming units, microbial growth, pellicle, supragingival

## Abstract

**Objectives:**

Oral biofilms initiate with the formation of an acquired pellicle on dental surfaces, a thin layer of salivary glycoproteins that provides a substrate for microbial adhesion. This study aimed to assess the necessity of a preformed pellicle for biofilm growth in vitro by analyzing the development of a standardized six‐species biofilm, comprising *Actinomyces oris*, *Veillonella dispar*, *Fusobacterium nucleatum*, *Streptococcus sobrinus*, *Streptococcus oralis*, and *Candida albicans*.

**Materials and Methods:**

Biofilms were cultivated on bovine enamel discs under two conditions: (1) precoated with human saliva to simulate a pellicle and (2) without a preformed pellicle. Colony‐forming units (CFUs) of each microbial species were quantified after incubation in either human saliva or a NaCl‐based medium at 16 and 64 h.

**Results:**

The analysis revealed no significant differences in CFU counts between discs with or without a preformed pellicle, regardless of whether biofilms were grown in human saliva or NaCl medium, with one exception: S. oralis in pellicle/NaCl (6.7 Log_10_) medium at 16 h showed a slight decrease in the absence of a pellicle (5.9 Log_10_).

**Conclusions:**

These findings suggest that microbial adhesion and subsequent biofilm development occurred independently of an initial pellicle. The preformed salivary pellicle does not seem to play a significant role in the initial development of this in vitro biofilm model. Biofilm testing in laboratory settings, especially for studies on antimicrobial efficacy, could be simplified, as pellicle formation may not be an essential requirement. Although no significant differences in biofilm development were observed between pellicle and no‐pellicle conditions, the growth medium may have influenced pellicle interactions, warranting further investigation of media effects on pellicle formation. Existing assumptions about pellicle dependence in biofilm formation are challenged, and suggest that in vitro models without a pellicle may still provide valid platforms for studying biofilms and testing antimicrobial agents effectively.

## 1 Introduction

Bacterial biofilms consist of a well‐established network of “aggregates of microorganisms,” which are tightly embedded in a self‐produced matrix of extracellular polymeric substances (EPS), enabling the bacterial cells to cohesion to each other and/or adhesion to external surfaces (Vert et al. 2012; Flemming et al. 2016). The microbial cells in a biofilm have a survival advantage over planktonic bacterial cells, owing mainly to the properties of the EPS matrix, which provide proximity for intercellular interactions within a biofilm, and an altered expression of genes (Flemming et al. 2016; Stoodley et al. 2002; Flemming and Wingender 2010). The highly complex and heterogeneous EPS‐matrix comprises polysaccharides, proteins, lipids, and nucleic acids (Cieplik et al. 2022; Bowen et al. 2018; Jakubovics et al. 2021). It serves to protect microorganisms against extreme environmental conditions, antimicrobial substances, and host immune system while also enabling nutrient acquisition (Flemming et al. 2016; Flemming and Wingender 2010; Rath et al. 2021).

The original five‐step model of biofilm formation was based on investigations in *Pseudomonas aeruginosa*, which described the initiation of biofilms by single planktonic cells, attachment to suitable surfaces, progression to mature biofilms, and finally the dispersion of bacterial cell populations (Sauer et al. 2002) and reviewed in Sauer et al. (2022). The crucial starting point in the development of oral biofilms is indeed the formation of a pellicle on dental surfaces. Pellicle was first described by Alexander Nasmyth in 1839 as a “persistent dental capsule” (Lendenmann et al. 2000; Nasmyth 1839). Further research established the enamel pellicle as an acellular, bacteria‐free membrane (Dawes 1963), which is acquired on exposed, freshly cleaned dental surfaces as early as 1 min after exposure (Hannig 1999), and establishes itself as a 30–100 nm layer comprising proteins, carbohydrates, and glycolipids (Lendenmann et al. 2000; Hannig and Bössmann 1989; Chawhuaveang et al. 2021; Rehage et al. 2017).

A spatiotemporal model of oral biofilm formation has been described (Kolenbrander et al. 2002; Kolenbrander and London 1993), in which initially the early colonizers bind to receptors in the acquired pellicle. Some early colonizers include *Streptococcus* spp., *Actinomyces* spp., and *Capnocytophaga* spp. As the biofilm grows, late colonizers, such as *Prevotella intermedia*, *Porphyromonas gingivalis*, *Aggregatibacter actinomycetemcomitans*, and *Treponema* spp., bind via *Fusobacterium nucleatum* to the previously bound early colonizers, leading to co‐aggregation of multiple bacterial species and establishing a complex network that favors the growth of biofilm (Kolenbrander et al. 2002; Kolenbrander and London 1993; Parashar et al. 2015). During the process of biofilm formation, bacterial cells continuously synthesize an exopolysaccharide matrix (Mihai et al. 2015). The study of oral biofilms is essential since they have a detrimental impact on oral diseases, such as dental caries, periodontitis, periimplantitis, and endodontic infections (reviewed by Abebe 2021). Oral biofilms can impede successful therapy using antibiotics and antimicrobial mouth rinses (reviewed by Rath et al. 2021).

Numerous multispecies models have been developed for the study of dental plaque, microbial adhesion to solid substrates, biofilm permeability, and the effectiveness of chemical agents against plaque elimination. Some of these models are based on a flow cell system for bacterial attachment and detachment, growth, and susceptibility testing (Christersson et al. 1987; Larsen and Fiehn 1995). Others employ a chemostat system to investigate biofilm development and assess the efficacy of antimicrobials against plaque (Bowden 1999; Bradshaw et al. 1996; Herles et al. 1994; Kinniment et al. 1996). These methods offer advantages in studying factors that affect microbial cell adhesion and detachment. However, they come with certain drawbacks, including the need for extended maintenance, the use of large volumes, which can be inefficient when working with smaller samples, and the impracticality for testing compounds during short exposure times, among other limitations.

Therefore, a new biofilm model, known as the “Zurich biofilm model,” was developed and described by Guggenheim et al. (2001). This model focused on the study of supragingival species and aimed to provide a technically simple approach to analyze *Actinomyces naeslundii*, *Veillonella dispar*, *Fusobacterium nucleatum*, *Streptococcus sobrinus*, and *Streptococcus oralis*. It allowed for the determination of biofilm composition and testing of antimicrobials at specific time points with high reproducibility (Guggenheim et al. 2001). To better simulate *in vivo* conditions in the oral environment, the model was further modified to include a yeast, *Candida albicans (*Shapiro et al. 2002; Ammann et al. 2012; Thurnheer and Paqué 2021). In this approach, microbial cells are cultivated anaerobically in a saliva‐based medium on either hydroxyapatite discs or bovine enamel discs coated with a salivary pellicle (Guggenheim et al. 2001; Shapiro et al. 2002; Guggenheim et al. 2004).

The aim of the present study was to utilize the Zurich biofilm model and analyze the growth of the six aforementioned microbial species (comprising five bacterial species and one yeast) both with and without the presence of a salivary pellicle. While the acquired pellicle is traditionally viewed as essential for the initial attachment of bacteria and the formation of biofilm on dental surfaces, this study aims to investigate whether a preformed pellicle is indeed necessary for establishing a supragingival biofilm in an in vitro model.

## 2 Materials and Methods

### 2.1 Preparation of Microbial Cultures

The Zurich biofilm multispecies model, which analyzes bacterial species found in supragingival plaque, was initially developed by Guggenheim et al. (2001). This model was subsequently expanded to include *C. albicans* (Shapiro et al. 2002; Ammann et al. 2012; Thurnheer and Paqué 2021). The methodology used in this study is based on the materials and methods outlined in the initial publication of the Zurich biofilm model by Guggenheim et al. (2001). The following microbial strains were utilized in this study: *A. oris* (OMZ 745; formerly known as *A. naeslundii*), *V. dispar* (OMZ 493), *F. nucleatum* (OMZ 598), *S. mutans* (OMZ 918), *S. oralis* (OMZ 607), and *C. albicans* (OMZ 110).

The strains were cultivated on Columbia blood agar (CBA; Becton, Dickinson and Company, Sparks, MD, USA) plates for 48 h at 37°C under anaerobic conditions (10% CO_2_ was additionally provided for *C. albicans*), followed by transfer to a liquid growth medium containing filter‐sterilized fluid universal medium (FUM), supplemented with 67 mmol/L Sorensen's buffer, pH 7.2 (modified FUM; mFUM) and 0.3% glucose and incubation under anaerobic conditions for 12 h (Gmür and Guggenheim 1983). For the cultivation of *V. dispar*, the medium was additionally supplemented with 1% (w/v) Na‐lactate. The cultures were further diluted 1:10 in the liquid growth medium and cultured to achieve an exponential growth phase (approximately 7 h or until OD_550_ = 1 was reached). A volume of 200 µL of this culture was used as inoculum as described in the experimental setup in Section 2.3. In parallel, an analysis of the inocula was performed by plating a series of culture dilutions on agar plates containing the respective growth medium to ensure optimal growth and colony formation. The following selective agar plates were used: Columbia Blood Agar plate (CBA base: Oxoid CM331, Thermofischer, UK; supplemented with 5% (v/v) hemolyzed human blood) for all microorganisms; Mitis‐Salivarius agar plate (MSAT base: Difco 0298‐17‐2, USA + 0.001% (w/v) Na tellurite) for *S. mutans* and *S. oralis*; Fastidious anaerobic agar plate (FAA base 7621 Biologische Arbeitsgemeinschaft GmbH, Germany, + 1 mg/L erythromycin, Sigma + 4 mg/L vancomycin, Eli Lilly, Switzerland + 1 mg/L norfloxacin, Sigma) for *F. nucleatum*; and Biggy agar plate (Difco 0635‐17‐4, USA) for *C. albicans*.

### 2.2 Saliva

Human saliva was collected in the Center of Dental Medicine, Zurich, Switzerland, throughout 1 h using sterile tubes; a gap of at least 1.5 h was ensured post‐meals and teeth cleaning to ensure saliva purity. Subsequently, the collected saliva samples were pooled and centrifuged at 27,500 × *g*, 4°C for 30 min. The resulting supernatant was then pasteurized at 60°C for 30 min, followed by centrifugation at 27,500 × *g*, 4°C for 30 min. The resulting supernatant was transferred into sterile tubes and stored at −20°C until use. The volunteers did not have any systemic or oral diseases and abstained from using antibiotics or local antimicrobials for 3 months prior to sampling.

### 2.3 Experimental Setup

The experiments were carried out in 24‐well tissue culture plates. After pellicle and biofilm formation, the bovine enamel discs were incubated in either human saliva or 0.9% NaCl.

#### 2.3.1 Pellicle Formation

A volume of 800 μL of the human saliva and 0.9% NaCl was pipetted into the 24‐well tissue culture plates. Before the addition, the human saliva was diluted 1:2 in 0.225% NaCl. In each well, one randomly selected bovine enamel disc was placed, and the plates were incubated under constant shaking at 95 rpm, RT for 4 h to allow pellicle development. The bovine discs were sourced from a slaughterhouse. The dentin samples underwent pretreatment using an automated grinding machine (Tegramin‐30, Stuers) set to 5 N and 80 rpm, initially utilizing 2000‐grit carborundum paper for 20 s, followed by 4000‐grit paper for an additional 30 s.

#### 2.3.2 Biofilm Formation

Biofilm formation was carried out in 24‐well tissue culture plates. To each well, 1120 µL of either human saliva or 0.9% NaCl and 480 µL of mFUM supplemented with 0.3% (w/v) glucose were added. The plates were equilibrated at 37°C for 45 min. The bovine enamel discs, which were previously used for pellicle formation, were now carefully transferred to these new plates. Biofilm formation was initiated by adding 200 µL of the previously prepared inocula (Section 2.1) to the enamel discs and was defined as the 0 h time point. The cell culture plates were then incubated at 37°C under anaerobic conditions, followed by sample collection and analysis after 16 and 64 h. Each combination of pellicle and medium was tested in triplicates. For each analysis and time point, the experiment was conducted thrice, resulting in a total of nine values per test group.

#### 2.3.3 Harvesting of Biofilms and Analysis of Colony‐Forming Units (CFUs)

The first washing steps and medium change were performed after 16 h of incubation in the anaerobic chamber. For this purpose, the biofilms were washed thrice by gentle dipping in 2 mL 0.9% NaCl solution, followed by transfer to a new 24‐well tissue culture plate containing 1120 µL of either human saliva or 0.9% NaCl and 480 µL of mFUM medium supplemented with 0.15% (w/v) glucose and 0.15% (w/v) sucrose, instead of 0.3% (w/v) glucose (Guggenheim et al. 2001). The biofilms were washed again after 20, 24, 40, 44, 48, and 64 h as described above; however, a medium change was carried out only at the 40 h time point. During the entire duration of the experiment and immediately after each medium change, the plates were incubated in the anaerobic chamber at 37°C.

Harvesting of biofilms from the bovine enamel discs was performed at 16 and 64 h as follows: the discs were first washed thrice in 0.9% NaCl as described above and then individually placed in tubes containing 1 mL 0.9% NaCl solution. From this point on, the tubes were kept on ice. The tubes were then vortexed for 2 min to detach the biofilms from the discs, followed by disc removal and gentle sonication of the NaCl solution (now containing the biofilms) at 30 W for 3 s to disrupt the cell aggregates.

The contents of the tubes were diluted using 0.9% NaCl, and serial dilutions were plated on the various agar plates, as described in Section 2.1. CBA‐ and FAA plates were incubated anaerobically, MTA plates and Biggy plates were incubated aerobically (+10% CO_2_), at 37°C, and colonies were counted after 72 h. Colony counting and analysis were performed based on the morphology of each colony by comparing it to the colonies on the inoculum plates. For colonies with unclear morphology, an Olympus microscope at a magnification of 2–10× was used.

### 2.4 Statistics

Statistical analysis was performed with the Mann–Whitney test using GraphPad Prism 9 software (GraphPad Software Inc., La Jolla, California) to compare independent groups directly. Bonferroni correction was applied to account for the multiple pairwise comparisons performed during the Mann–Whitney test analysis. A 95% confidence interval was used.

## 3 Results

The primary objective of our study was to analyze differences in CFUs on enamel discs containing a pellicle in comparison to discs without one. This objective was assessed after 16 and 64 h of incubation of the discs with the five bacterial species and *C. albicans*.

### 3.1 Biofilm Analysis After 16 h of Incubation

**FIGURE 1:**
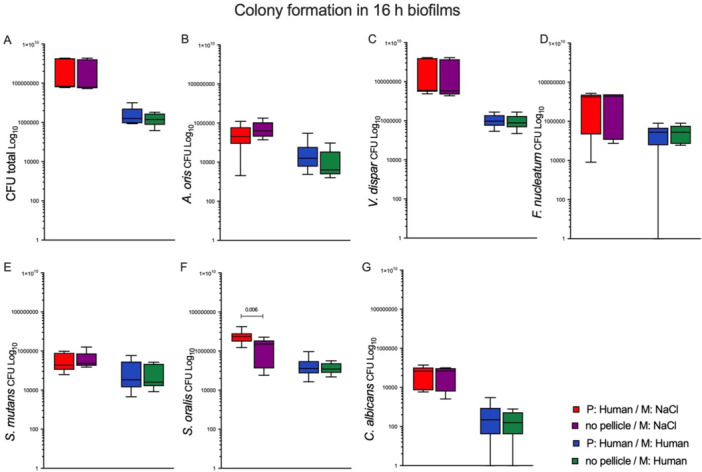
Colony‐forming units (CFUs) on bovine enamel discs post‐incubation for 16 h in the absence or presence of a pellicle from human saliva. The bovine enamel discs, consisting of a pellicle, were incubated in a medium comprising either 0.9% NaCl or human saliva. Additionally, discs without a previously formed pellicle were incubated in 0.9% NaCl and human saliva and served as controls. Following incubation, the colonies were harvested, serially diluted, plated on respective growth media, and counted based on their morphology. Box plots depict colonies for (A) CFU total, (B) *A. oris*, (C) *V. dispar*, (D) *F. nucleatum*, (E) *S. mutans*, (F) *S. oralis*, and (G) *C. albicans*. Statistical analysis was performed with the Mann–Whitney test using GraphPad Prism 9. The statistical significance based on a 95% confidence interval is depicted above each box as follows: ****p < 0.0001, ***p < 0.001, **p < 0.01, *p < 0.05, ns = not significant. The experiment was conducted thrice for each analysis, resulting in a total of nine values per test group.

Figure 1 presents the results of the biofilm analysis after 16 h of incubation. A significant finding emerged when comparing the total CFUs of microbes on enamel discs with pellicles derived from human saliva versus those without pellicles grown in a human medium. Notably, the two groups exhibited no statistically significant difference, indicating that the presence of saliva pellicles may not play as crucial a role in microbial colonization as previously assumed. Similarly, the total CFUs of microbes grown in NaCl medium, whether in the presence or absence of a pellicle formed from human saliva, also showed no differences (Figure 1).

A detailed analysis of individual CFUs of five bacterial species, along with *C. albicans*, in both media (with human saliva and NaCl) revealed no statistically significant differences when comparing growth in the presence or absence of a human saliva pellicle (Figure 1). However, one exception was noted for *S. oralis*, which demonstrated statistically significantly higher CFUs on enamel discs with a human pellicle (Figure 1). This suggests that while most microbial species may not be heavily influenced by human saliva pellicles, *S. oralis* may have a unique interaction that warrants further investigation.

After thoroughly analyzing the groups cultured with and without the human pellicle, as well as in different growth media (human saliva or NaCl), some noteworthy observations were made: all microbial species exhibited higher CFUs in the NaCl medium compared to those cultured in human saliva (Figure 1). This finding underscores the importance of nutrient‐rich environments in supporting microbial growth, potentially influencing strategies for oral health management.

### 3.2 Biofilm Analysis After 64 h of Incubation

**FIGURE 2:**
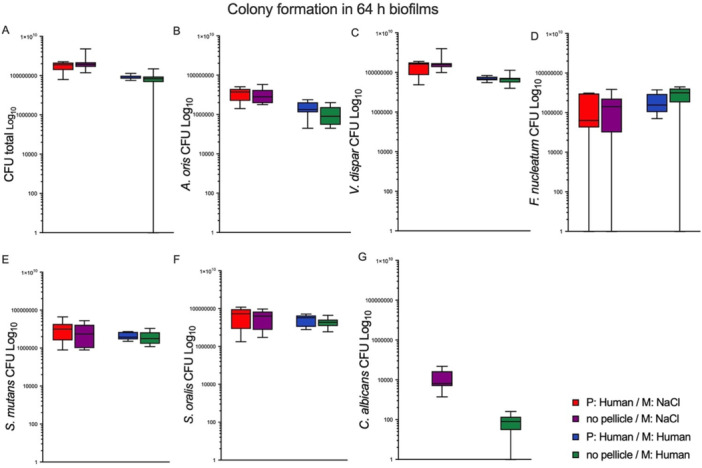
Colony‐forming units (CFUs) on bovine enamel discs post‐incubation for 64 h in the absence or presence of a pellicle from human saliva. The bovine enamel discs, consisting of a pellicle, were incubated in a medium comprising either 0.9% NaCl or human saliva, as shown in the Figure. Additionally, discs without a previously formed pellicle were incubated in 0.9% NaCl and human saliva and served as controls. Following incubation, the colonies were harvested, serially diluted, plated on respective growth media, and counted based on their morphology. Box plots depict colonies for (A) CFU total, (B) *A. oris*, (C) *V. dispar*, (D) *F. nucleatum*, (E) *S. mutans*, (F) *S. oralis*, and (G) *C. albicans*. Statistical analysis was performed with the Mann–Whitney test using GraphPad Prism 9. The statistical significance based on a 95% confidence interval is depicted above each box as follows: ****p < 0.0001, ***p < 0.001, **p < 0.01, *p < 0.05, ns = not significant. The experiment was conducted thrice for each analysis, resulting in a total of nine values per test group.

The analysis results of bovine enamel discs after a 64‐h incubation period are shown in Figure 2. No significant differences were found in the total CFUs or among individual bacterial strains, including *C. albicans*, when comparing human medium with and without a pellicle. Additionally, pellicle formation did not significantly impact the total CFUs or individual CFUs of bacterial and fungal strains in NaCl medium (see Figure 2).

These findings strongly suggest that the presence of a pellicle does not play a critical role in modulating microbial growth under the conditions tested. This lack of significant impact raises important questions about the traditional views regarding pellicle formation and its role in oral microbiology.

## 4 Discussion

The purpose of the present study was to analyze the effect of a pellicle on microbial colony formation in vitro using the Zurich multispecies Biofilm model. We aimed to study the growth of five bacterial and one fungal species after 16 and 64 h of biofilm formation about the presence of a pellicle and compare it with colony growth on bovine enamel discs without a pellicle.

An important observation at both incubation time points, 16 and 64 h, is obtained by comparison of colony growth on discs that are different only in the pellicle (human or none) but were incubated in the same medium (either human or NaCl). In both growth media, the pellicle did not influence microbial growth (total CFUs and individual strains, except *S. oralis* at 16 h, Figure 2). Some studies suggest that microbial adherence and biofilm formation are not necessarily dependent on a pellicle. Larsen et al. established a model based on a flow method for biofilm formation and susceptibility testing, in which the authors investigated CFUs about the presence and absence of a pellicle from human saliva (Larsen and Fiehn 1995). The researchers discovered that the average number of *Streptococcus sanguis* CFUs was higher on surfaces coated with saliva as opposed to PBS. However, this difference was not statistically significant. This suggests that while a pellicle may enhance bacterial adhesion, it is not always necessary for initial microbe binding (Larsen and Fiehn 1995). Moreover, oral bacteria are capable of adhering to intraorally placed artificial tooth surfaces (plastic films) without any previously formed layer of pellicle (Rönström et al. 1975; Brecx et al. 1980, 1981). While CFU analysis provides valuable quantitative data on microbial viability, future studies utilizing advanced imaging techniques and biochemical assays are necessary to comprehensively validate the observed independence of biofilm development from initial pellicle formation.

In our groups without a pellicle, the biofilm formation in a medium containing either human saliva or NaCl was initiated without a preformed pellicle. It is, however, difficult to exclude that during the incubation, the formation of a pellicle preceded the bacterial attachment and growth and, therefore, could be a reason for similar results, as when a preformed pellicle was present. Since adherence of proteins and pellicle formation can occur as soon as 1 min after teeth cleaning (Hannig 1999), this presumption seems quite plausible. Very interestingly, as reviewed by (Gristina et al. 1989), bacterial adhesion to implant surfaces may either occur via direct interaction of bacteria with the substratum or via adsorbed glycoproteins. On a substratum, such as newly implanted devices, microbial cells approach the surface and compete with the host tissue cells in binding to the surface. However, due to similar surface charges of microbes and substratum, they tend to repel each other. Although a close apposition of microbes to the substratum takes place, it is conceivable that adhesion and concomitant aggregation occur via adsorbed host glycoproteins; thus, it is likely that in our in vitro model, adsorption of glycoproteins proved to be the “winners of the race” for the enamel disc surface (Gristina et al. 1989; Hannig et al. 2007). Since the physicochemical interactions responsible for binding salivary proteins to the enamel surface lead to a gain in entropy, these are likely to contribute to the initial steps of interactions taking place on a dental surface (Hannig and Joiner 2006; Vassilakos et al. 1993, 1992). In our study, we acknowledge the importance of detecting pellicle formation on enamel discs, which is crucial for understanding the initial stages of biofilm development. While the methodology involved incubating bovine enamel discs in human saliva to allow pellicle formation, detecting its presence can be challenging. Several techniques can be employed to assess pellicle formation effectively. One such method is atomic force microscopy, which provides high‐resolution images to evaluate surface topography and can quantify changes linked to protein adsorption, indicating pellicle development (Wang et al. 2022). Additionally, optical techniques, such as ellipsometry or reflectometry, can be utilized to analyze the thickness and composition of the pellicle layer formed on the enamel surface. Incorporating these detection methods in future studies would provide a more comprehensive understanding of the pellicle's role and its development on enamel surfaces, further validating our findings about the absence or presence of pellicle in different conditions.

The decision to use bovine enamel discs in our study was based on several practical and scientific considerations. First, bovine enamel is readily available and cost‐effective compared to human enamel, making it a practical choice for research involving large sample sizes (Ayoub et al. 2020). Notably, previous studies have shown that the chemical and structural properties of bovine enamel are quite similar to those of human enamel, allowing for comparable results in biofilm formation studies (Ran et al. 2014; Azevedo et al. 2011). Furthermore, using bovine enamel enables standardization across experiments, as it can be obtained from the same source with consistent properties, unlike human samples that may exhibit considerable variability due to factors like age, diet, and oral hygiene practices (Ayoub et al. 2020).

Reflecting on the implications of our findings, it is pertinent to consider the potential effects of using synthetic hydroxyapatite discs as an alternative to bovine enamel. The surface chemistry of the substrate can significantly influence pellicle adsorption and bacterial attachment. Numerous studies have demonstrated that the electrostatic interactions between salivary proteins and hydroxyapatite surfaces can differ based on the material's composition and characteristics (Baumann et al. 2015). Hydrophobic and electrostatic properties play crucial roles in determining how salivary components adsorb and subsequently affect microbial adherence (Joiner et al. 2004). Given that synthetic hydroxyapatite discs can create a more controlled environment for studying these interactions, utilizing this material might yield similar results regarding biofilm formation dynamics, albeit with potential differences rooted in surface chemistry.

A notable strength of the present study lies in the use of the Zurich biofilm model, an accepted model for effectively evaluating the in vitro impact of interventions such as mouth rinses. This model allows for evaluating antibacterial solutions within limited timeframes, as demonstrated in previous studies (Larsen and Fiehn 1995). This capability provides a relevant link between outcomes from various in vitro experiments involving mouth rinses and subsequent clinical observations (Guggenheim et al. 2004). To simulate the influence of natural salivary flow, the model includes a rinse, thrice daily, with a physiological NaCl solution (Guggenheim et al. 2004). This approach improves the ability of the model to adequately simulate conditions in the oral cavity. While our approach may not isolate the role of the pellicle in bacterial attachment, it reflects conditions where the pellicle is likely present and can influence biofilm formation.

The presence of human saliva in the inoculum introduces additional organic components, such as salivary proteins, glycoproteins, and enzymes. These components may compete with bacterial adhesins for binding sites on the enamel surface, potentially reducing bacterial attachment and resulting in lower CFU counts compared to NaCl/mFUM, which lacks these competing components. Additionally, saliva contains antimicrobial peptides and enzymes, like lysozyme and lactoferrin, that could inhibit bacterial growth or attachment in the saliva/mFUM condition. Finally, human saliva may promote the development of a different biofilm community or delay biofilm maturation compared to NaCl/mFUM, which could lead to lower overall CFU counts in the conditions studied.

Our primary aim was to evaluate the role of the pellicle in the growth of a defined six‐species biofilm, which includes early colonizers such as *Streptococcus oralis* and *Actinomyces oris*, one bridge organism (*Fusobacterium nucleatum*), and *Candida albicans* to better simulate in vivo conditions. While we acknowledge the importance of including additional secondary colonizers like *Porphyromonas gingivalis* and *Tannerella forsythia* in the maturation of oral biofilms, our selection was influenced by the need for a simplified and reproducible model to address the specific research question of whether a pellicle is necessary for biofilm formation. Including more secondary colonizers in future studies could provide a more comprehensive understanding of the ecological succession and complexity of oral biofilms.

However, it is essential to acknowledge a limitation inherent to all biofilm models, which concerns the limited range of bacterial strains included. While the model provides controlled conditions for examining the role of the pellicle, it may not fully replicate the complex dynamics of microbial suspension and biofilm formation in the mouth. With approximately 700 bacterial species, the human oral microbiota is highly diverse (Aas et al. 2005). It is noteworthy that the Zurich biofilm model, as mentioned previously, considers only six species. Although this selection offers valuable insights, the broader range of the oral microbiota should be taken into account when interpreting the results. The extended exposure period was selected to simulate clinical conditions where biofilms can develop over time due to constant exposure to saliva and dietary components. However, we recognize that this setup may limit our ability to detect more subtle early‐stage differences that could be affected by the presence or absence of the pellicle. Although our static, horizontal disc model offers valuable insights into pellicle‐mediated biofilm formation in a controlled setting, it does not fully replicate the dynamic, fluid‐driven environment of the oral cavity, in which bacteria are periodically exposed to saliva and growth medium. While our study provides initial evidence challenging the necessity of salivary pellicle for biofilm formation, further investigations utilizing diverse methodologies are essential to substantiate these findings and elucidate the complex interactions among oral microorganisms.

Considering the broader impact of our findings, it is essential to note that the necessity of a preformed salivary pellicle may also be re‐evaluated in other multispecies biofilm models, both static and dynamic. For instance, models that include species like *S. mutans*, *Streptococcus gordonii*, and *Streptococcus sanguinis* have demonstrated that biofilm composition can significantly influence the overall interactions and development within the community, suggesting that the absence of a preformed pellicle still allows for effective biofilm establishment (Yan et al. 2023; Jiang et al. 2018). Additionally, research indicates that dynamic models, which better simulate physiological conditions, can result in diverse microbial responses and arrangements that challenge traditional assumptions regarding pellicle dependency in biofilm formation (Blanc et al. 2014; Park et al. 2014; Rath et al. 2017). Notably, alternative biofilm models could have been utilized to achieve the aims of this study, such as single‐species biofilms or different multispecies arrangements. However, we assert that the Zurich model was a particularly suitable choice for this investigation due to its established relevance in simulating oral biofilm dynamics and its capacity to recreate the complexities of supragingival biofilm formation. The Zurich model effectively allows for the incorporation of key microbial species that interact in the oral cavity and provides a standardized approach to study biofilm development under controlled conditions. The impact of the culture medium on biofilm development cannot be understated, as variations in nutrient composition can significantly influence microbial growth, interaction dynamics, and overall experimental outcomes.

This study's findings are constrained by potential limitations in microbial diversity and the static nature of the model employed. The use of a static model may not accurately represent the dynamic interactions and competitive behaviors observed in natural biofilms, which often feature a broader diversity of microbial species engaging in complex interrelationships. Furthermore, the reliance on a specific microbial community composition may limit the generalizability of our results to other contexts where different microbial interactions occur (Brockmann et al. 2013).

In summary, the results indicate that the acquired pellicle does not significantly affect microbial adhesion, as shown by the in vitro measurements of the current study. However, careful interpretation of this conclusion is warranted because of the limitations associated with the chosen experimental setup. The *in vitro* model used in this study is by far not a complete representation of the diverse oral microbiota found *in vivo*, which could potentially lead to deviating results when extrapolated to real situations. However, based on the observations in our study, it can be concluded that future studies using the Zurich biofilm model may eliminate the need for prior pellicle formation before microbial inoculation. Future research should also consider a broader spectrum of microbial species and the incorporation of varied environmental conditions to enhance our understanding of oral biofilm formation dynamics. Finally, it is important to emphasize that while we did not observe significant differences in biofilm development between the preformed pellicle and no‐pellicle conditions, we acknowledge that the composition of the growth medium could have facilitated pellicle precursor interactions. Therefore, future research should explore the potential effects of various media compositions on pellicle formation dynamics.

## Author Contributions

Shengjile Deari and Monika Gothwal interpreted the data and drafted the manuscript. Kay Gränicher contributed to data acquisition and data analysis. Thomas Thurnheer conceptualized the study and analyzed the data. Lamprini Karygianni and Thomas Attin contributed to manuscript correction and data interpretation. All authors read and approved the final manuscript.

## Conflicts of Interest

The authors declare no conflicts of interest.

## Disclosure/Publisher's Note

The statements, opinions, and data contained in all publications are solely those of the individual author(s) and contributor(s) and not of MDPI and/or the editor(s). MDPI and/or the editor(s) disclaim responsibility for any injury to people or property resulting from any ideas, methods, instructions, or products referred to in the content.

## Data Availability

The data that support the findings of this study are available from the corresponding author upon reasonable request.

## References

[cre270276-bib-0001] Aas, J. A. , B. J. Paster , L. N. Stokes , I. Olsen , and F. E. Dewhirst . 2005. “Defining the Normal Bacterial Flora of the Oral Cavity.” Journal of Clinical Microbiology 43, no. 11: 5721–5732. 16272510 10.1128/JCM.43.11.5721-5732.2005 PMC1287824

[cre270276-bib-0002] Abebe, G. M. 2021. “Oral Biofilm and Its Impact on Oral Health, Psychological and Social Interaction.” International Journal of Oral Health Dentistry 7: 127. 10.23937/2469-5734/1510127.

[cre270276-bib-0003] Ammann, T. W. , R. Gmür , and T. Thurnheer . 2012. “Advancement of the 10‐Species Subgingival Zurich Biofilm Model by Examining Different Nutritional Conditions and Defining the Structure of the In Vitro Biofilms.” BMC Microbiology 12: 227. 23040057 10.1186/1471-2180-12-227 PMC3561252

[cre270276-bib-0004] Gristina, A. G. , P. T. Naylor , and Q. Myrvik . 1989. “The Race for the Surface: Microbes, Tissue Cells, and Biomaterials.” In Molecular Mechanisms of Microbial Adhesion edited by L. Switalski , M. Höök , E. Beachey . Springer. 10.1007/978-1-4612-3590-3_15.

[cre270276-bib-0005] Ayoub, H. M. , R. L. Gregory , Q. Tang , and F. Lippert . 2020. “Comparison of Human and Bovine Enamel in a Microbial Caries Model at Different Biofilm Maturations.” Journal of Dentistry 96: 103328. 32240676 10.1016/j.jdent.2020.103328

[cre270276-bib-0006] Azevedo, M. S. , F. H. van de Sande , A. R. Romano , and M. S. Cenci . 2011. “Microcosm Biofilms Originating From Children With Different Caries Experience Have Similar Cariogenicity Under Successive Sucrose Challenges.” Caries Research 45, no. 6: 510–517. 21967836 10.1159/000331210

[cre270276-bib-0007] Baumann, T. , T. S. Carvalho , and A. Lussi . 2015. “The Effect of Enamel Proteins on Erosion.” Scientific Reports 5: 15194. 26468660 10.1038/srep15194 PMC4606565

[cre270276-bib-0008] Blanc, V. , S. Isabal , M. C. Sánchez , et al. 2014. “Characterization and Application of a Flow System for in Vitro Multispecies Oral Biofilm Formation.” Journal of Periodontal Research 49, no. 3: 323–332. 23815431 10.1111/jre.12110

[cre270276-bib-0009] Bowden, G. H. 1999. “Controlled Environment Model for Accumulation of Biofilms of Oral Bacteria.” Methods in Enzymology 310: 216–224. 10547795 10.1016/s0076-6879(99)10019-3

[cre270276-bib-0010] Bowen, W. H. , R. A. Burne , H. Wu , and H. Koo . 2018. “Oral Biofilms: Pathogens, Matrix, and Polymicrobial Interactions in Microenvironments.” Trends in Microbiology 26, no. 3: 229–242. 29097091 10.1016/j.tim.2017.09.008 PMC5834367

[cre270276-bib-0011] Bradshaw, D. J. , P. D. Marsh , K. M. Schilling , and D. Cummins . 1996. “A Modified Chemostat System to Study the Ecology of Oral Biofilms.” Journal of Applied Bacteriology 80, no. 2: 124–130. 8642010 10.1111/j.1365-2672.1996.tb03199.x

[cre270276-bib-0012] Brecx, M. , A. Rönström , J. Theilade , and R. Attström . 1981. “Early Formation of Dental Plaque on Plastic Films. 2. Electron Microscopic Observations.” Journal of Periodontal Research 16, no. 2: 213–227. 6453984 10.1111/j.1600-0765.1981.tb00969.x

[cre270276-bib-0013] Brecx, M. , J. Theilade , and R. Attström . 1980. “Influence of Optimal and Excluded Oral Hygiene on Early Formation of Dental Plaque on Plastic Films. A Quantitative and Descriptive Light and Electron Microscopic Study.” Journal of Clinical Periodontology 7, no. 5: 361–373. 6936411 10.1111/j.1600-051x.1980.tb02009.x

[cre270276-bib-0014] Brockmann, D. , A. Caylet , R. Escudié , J. P. Steyer , and N. Bernet . 2013. “Biofilm Model Calibration and Microbial Diversity Study Using Monte Carlo Simulations.” Biotechnology and Bioengineering 110, no. 5: 1323–1332. 23280411 10.1002/bit.24818

[cre270276-bib-0015] Chawhuaveang, D. D. , O. Y. Yu , I. X. Yin , W. Y. H. Lam , M. L. Mei , and C. H. Chu . 2021. “Acquired Salivary Pellicle and Oral Diseases: A Literature Review.” Journal of Dental Sciences 16, no. 1: 523–529. 33384841 10.1016/j.jds.2020.10.007 PMC7770358

[cre270276-bib-0016] Christersson, C. E. , M. S. Fqrnalik , R. E. Baier , and P. O. J. Glantz . 1987. “In Vitro Attachment of Oral Microorganisms to Solid Surfaces: Evaluation of a Controlled Flow Method.” European Journal of Oral Sciences 95, no. 2: 151–158. 10.1111/j.1600-0722.1987.tb01823.x 3470908

[cre270276-bib-0017] Cieplik, F. , C. Aparicio , J. Kreth , and G. Schmalz . 2022. “Development of Standard Protocols for Biofilm‐Biomaterial Interface Testing.” JADA Foundational Science 1: 100008. 10.1016/j.jfscie.2022.100008 PMC13229039 42238662

[cre270276-bib-0018] Dawes, C. 1963. “The Nomenclature of the Integuments of the Enamel Surface of Tooth.” British Dental Journal 115: 65–68.

[cre270276-bib-0019] Flemming, H.‐C. , and J. Wingender . 2010. “The Biofilm Matrix.” Nature Reviews Microbiology 8, no. 9: 623–633. 20676145 10.1038/nrmicro2415

[cre270276-bib-0020] Flemming, H.‐C. , J. Wingender , U. Szewzyk , P. Steinberg , S. A. Rice , and S. Kjelleberg . 2016. “Biofilms: An Emergent Form of Bacterial Life.” Nature Reviews Microbiology 14, no. 9: 563–575. 27510863 10.1038/nrmicro.2016.94

[cre270276-bib-0021] Gmür, R. , and B. Guggenheim . 1983. “Antigenic Heterogeneity of Bacteroides Intermedius as Recognized by Monoclonal Antibodies.” Infection and Immunity 42, no. 2: 459–470. 6196291 10.1128/iai.42.2.459-470.1983 PMC264452

[cre270276-bib-0022] Guggenheim, B. , E. Giertsen , P. Schüpbach , and S. Shapiro . 2001. “Validation of an In Vitro Biofilm Model of Supragingival Plaque.” Journal of Dental Research 80, no. 1: 363–370. 11269730 10.1177/00220345010800011201

[cre270276-bib-0023] Guggenheim, B. , M. Guggenheim , R. Gmür , E. Giertsen , and T. Thurnheer . 2004. “Application of the Zürich Biofilm Model to Problems of Cariology.” Caries Research 38, no. 3: 212–222. 15153691 10.1159/000077757

[cre270276-bib-0024] Hannig, C. , M. Hannig , O. Rehmer , G. Braun , E. Hellwig , and A. Al‐Ahmad . 2007. “Fluorescence Microscopic Visualization and Quantification of Initial Bacterial Colonization on Enamel In Situ.” Archives of Oral Biology 52, no. 11: 1048–1056. 17603998 10.1016/j.archoralbio.2007.05.006

[cre270276-bib-0025] Hannig, M. 1999. “Ultrastructural Investigation of Pellicle Morphogenesis at Two Different Intraoral Sites During a 24‐h Period.” Clinical Oral Investigations 3, no. 2: 88–95. 10803117 10.1007/s007840050084

[cre270276-bib-0026] Hannig, M. , and K. Bössmann . 1989. “Dental Pellicle (1). Ultrastructural Variety as Expression of Complex Formation and Maturation.” Die Quintessenz 40, no. 7: 1319–1327. 2640006

[cre270276-bib-0027] Hannig, M. , and A. Joiner . 2006. “The Structure, Function and Properties of the Acquired Pellicle.” Monographs in Oral Science 19: 29–64. 16374028 10.1159/000090585

[cre270276-bib-0028] Herles, S. , S. Olsen , J. Afflitto , and A. Gaffar . 1994. “Chemostat Flow Cell System: An In Vitro Model for the Evaluation of Antiplaque Agents.” Journal of Dental Research 73, no. 11: 1748–1755. 7983262 10.1177/00220345940730111101

[cre270276-bib-0029] Jakubovics, N. S. , S. D. Goodman , L. Mashburn‐Warren , G. P. Stafford , and F. Cieplik . 2021. “The Dental Plaque Biofilm Matrix.” Periodontology 2000 86, no. 1: 32–56. 33690911 10.1111/prd.12361 PMC9413593

[cre270276-bib-0030] Jiang, W. , Y. Wang , J. Luo , et al. 2018. “Effects of Antimicrobial Peptide GH12 on the Cariogenic Properties and Composition of a Cariogenic Multispecies Biofilm.” Applied and Environmental Microbiology 84, no. 24: e01423‐18. 10.1128/AEM.01423-18. 30341079 PMC6275336

[cre270276-bib-0031] Joiner, A. , D. Muller , U. M. Elofsson , and T. Arnebrant . 2004. “Ellipsometry Analysis of the In Vitro Adsorption of Tea Polyphenols Onto Salivary Pellicles.” European Journal of Oral Sciences 112, no. 6: 510–515. 15560834 10.1111/j.1600-0722.2004.00166.x

[cre270276-bib-0032] Kinniment, S. L. , J. W. T. Wimpenny , D. Adams , and P. D. Marsh . 1996. “Development of a Steady‐State Oral Microbial Biof ilrn Community Using the Constant‐Depth Film Ferrnenter.” Microbiology 142, no. Pt 3: 631–638. 8868438 10.1099/13500872-142-3-631

[cre270276-bib-0033] Kolenbrander, P. E. , R. N. Andersen , D. S. Blehert , P. G. Egland , J. S. Foster , and R. J. Palmer, Jr. 2002. “Communication Among Oral Bacteria.” Microbiology and Molecular Biology Reviews 66, no. 3: 486–505. 12209001 10.1128/MMBR.66.3.486-505.2002 PMC120797

[cre270276-bib-0034] Kolenbrander, P. E. , and J. London . 1993. “Adhere Today, Here Tomorrow: Oral Bacterial Adherence.” Journal of Bacteriology 175, no. 11: 3247–3252. 8501028 10.1128/jb.175.11.3247-3252.1993 PMC204720

[cre270276-bib-0035] Larsen, T. , and N. E. Fiehn . 1995. “Development of a Flow Method for Susceptibility Testing of Oral Biofilms In Vitro.” APMIS 103, no. 5: 339–344. 7654358 10.1111/j.1699-0463.1995.tb01117.x

[cre270276-bib-0036] Lendenmann, U. , J. Grogan , and F. G. Oppenheim . 2000. “Saliva and Dental Pellicle‐A Review.” Advances in Dental Research 14, no. 1: 22–28. 11842920 10.1177/08959374000140010301

[cre270276-bib-0037] Mihai, M. M. , A. M. Holban , C. Giurcăneanu , et al. 2015. “Microbial Biofilms: Impact on Pathogenesis of Periodontitis, Cystic Fibrosis, Chronic Wounds and Medical Device‐Related Infections.” Current Topics in Medicinal Chemistry 15, no. 16: 1552–1576. 10.2174/1568026615666150414123800. 25877092

[cre270276-bib-0038] Nasmyth, A. 1839. “On the Structure, Physiology, and Pathology of the Persistent Capsular Investments and Pulp of the Tooth.” Journal of the Royal Society of Medicine 22: 310–328. 10.1177/095952873902200123 PMC2116831 20895692

[cre270276-bib-0039] Parashar, A. , S. Parashar , A. Zingade , S. Gupta , and S. Sanikop . 2015. “Interspecies Communication in Oral Biofilm: An Ocean of Information.” Oral Science International 12, no. 2: 37–42.

[cre270276-bib-0040] Park, J. H. , J. K. Lee , H. S. Um , B. S. Chang , and S. Y. Lee . 2014. “A Periodontitis‐Associated Multispecies Model of An Oral Biofilm.” Journal of Periodontal & Implant Science 44, no. 2: 79–84. 24778902 10.5051/jpis.2014.44.2.79 PMC3999356

[cre270276-bib-0041] Ran, J. M. , C. C. Ieong , C. Y. Xiang , et al. 2014. “In Vitro Inhibition of Bovine Enamel Demineralization by Enamel Matrix Derivative.” Scanning 36, no. 2: 194–201. 23471716 10.1002/sca.21085

[cre270276-bib-0042] Rath, H. , S. N. Stumpp , and M. Stiesch . 2017. “Development of a Flow Chamber System for the Reproducible in Vitro Analysis of Biofilm Formation on Implant Materials.” PLoS One 12, no. 2: e0172095. 28187188 10.1371/journal.pone.0172095 PMC5302373

[cre270276-bib-0043] Rath, S. , S. C. B. Bal , and D. Dubey . 2021. “Oral Biofilm: Development Mechanism, Multidrug Resistance, and Their Effective Management With Novel Techniques.” Rambam Maimonides Medical Journal 12, no. 1: e0004. 33478627 10.5041/RMMJ.10428 PMC7835112

[cre270276-bib-0044] Rehage, M. , J. Delius , T. Hofmann , and M. Hannig . 2017. “Oral Astringent Stimuli Alter the Enamel Pellicle's Ultrastructure as Revealed by Electron Microscopy.” Journal of Dentistry 63: 21–29. 28619693 10.1016/j.jdent.2017.05.011

[cre270276-bib-0045] Rönström, A. , R. Attström , and J. Egelberg . 1975. “Early Formation of Dental Plaque on Platic Films. 1. Light Microscopic Observations.” Journal of Periodontal Research 10, no. 1: 28–35. 124332 10.1111/j.1600-0765.1975.tb00004.x

[cre270276-bib-0046] Sauer, K. , A. K. Camper , G. D. Ehrlich , J. W. Costerton , and D. G. Davies . 2002. “Pseudomonas Aeruginosa Displays Multiple Phenotypes During Development as a Biofilm.” Journal of Bacteriology 184, no. 4: 1140–1154. 11807075 10.1128/jb.184.4.1140-1154.2002 PMC134825

[cre270276-bib-0047] Sauer, K. , P. Stoodley , D. M. Goeres , et al. 2022. “The Biofilm Life Cycle: Expanding the Conceptual Model of Biofilm Formation.” Nature Reviews Microbiology 20, no. 10: 608–620. 35922483 10.1038/s41579-022-00767-0 PMC9841534

[cre270276-bib-0048] Shapiro, S. , E. Giertsen , and B. Guggenheim . 2002. “An in Vitro Oral Biofilm Model for Comparing the Efficacy of Antimicrobial Mouthrinses.” Caries Research 36, no. 2: 93–100. 12037365 10.1159/000057866

[cre270276-bib-0049] Stoodley, P. , K. Sauer , D. G. Davies , and J. W. Costerton . 2002. “Biofilms as Complex Differentiated Communities.” Annual Review of Microbiology 56, no. 1: 187–209. 10.1146/annurev.micro.56.012302.160705 12142477

[cre270276-bib-0050] Thurnheer, T. , and P. N. Paqué . 2021. “Biofilm Models to Study the Etiology and Pathogenesis of Oral Diseases.” Monographs in Oral Science 29: 30–37. 33427216 10.1159/000510197

[cre270276-bib-0051] Vassilakos, N. , T. Arnebrant , and P. O. Glantz . 1993. “An in Vitro Study of Salivary Film Formation at Solid/Liquid Interfaces.” European Journal of Oral Sciences 101, no. 3: 133–137. 10.1111/j.1600-0722.1993.tb01652.x 8322006

[cre270276-bib-0052] Vassilakos, N. , T. Arnebrant , J. Rundegren , and P. O. Glantz . 1992. “In Vitro Interactions of Anionic and Cationic Surfactants With Salivary Fractions on Well‐Defined Solid Surfaces.” Acta Odontologica Scandinavica 50, no. 3: 179–188. 1321547 10.3109/00016359209012761

[cre270276-bib-0053] Vert, M. , Y. Doi , K.‐H. Hellwich , et al. 2012. “Terminology for Biorelated Polymers and Applications (IUPAC Recommendations 2012).” Pure and Applied Chemistry 84, no. 2: 377–410.

[cre270276-bib-0054] Wang, R. , Y. Wang , Z. Lei , L. Hao , and L. Jiang . 2022. “Glucosyltransferase‐Modulated Streptococcus Mutans Adhesion to Different Surfaces Involved in Biofilm Formation by Atomic Force Microscopy.” Microbiology and Immunology 66, no. 11: 493–500. 36047500 10.1111/1348-0421.13025

[cre270276-bib-0055] Yan, Y. , H. Hailun , Y. Fenghui , et al. 2023. “Streptococcus Mutans dexA Affects Exopolysaccharides Production and Biofilm Homeostasis.” Molecular Oral Microbiology 38, no. 2: 134–144. 36270969 10.1111/omi.12395

